# Modular engineered outer membrane vesicles for synergistic cancer immunotherapy via PD-L1 blockade and epigenetic reprogramming

**DOI:** 10.3389/fimmu.2026.1915499

**Published:** 2026-07-31

**Authors:** Longxue Guan, Qinxiao Sun, Dandan Liang, Zhimin Li, Shiqi Zhang, Laili Huang, Xingang Guan, Hongxia Feng

**Affiliations:** 1 Department of Dermatology, The Affiliated Wenling Hospital of Taizhou University (The First People’s Hospital of Wenling), School of Medicine, Taizhou University, Taizhou, China; 2 College of Medical Technology, Beihua University, Jilin, China

**Keywords:** cancer immunotherapy, epigenetic regulation, OMVs, PD-L1 blockade, SpyCatcher/SpyTag

## Abstract

Immune checkpoint inhibitors have significantly improved therapeutic outcomes in advanced melanoma; however, their clinical efficacy is greatly limited by low overall response rates and the primary or acquired resistance. Here, we developed a SpyCatcher-engineered bacterial outer membrane vesicle (OMV) platform for the co-delivery of a PD-1/PD-L1 inhibitor, the D-PPA1 peptide, and the epigenetic regulator zebularine (Zeb). The resulting co-delivery system, OMV@PPA/Zeb, effectively blocked PD-1/PD-L1 interactions and markedly upregulated melanoma-associated antigens, including CD146 and TRP1, in melanoma cells. In a subcutaneous melanoma mouse model, OMV@PPA/Zeb substantially suppressed tumor growth without noticeable systemic toxicity. Moreover, OMV@PPA/Zeb significantly promoted the infiltration and activation of CD8^+^ T cells while reducing regulatory T cells (Tregs) within the tumor microenvironment. The potent antitumor immune response elicited by this platform contributed to its enhanced therapeutic efficacy against melanoma. Overall, this study provides a modular OMV-based strategy for combination immunotherapy in advanced tumors.

## 1 Introduction

Melanoma is one of the most aggressive skin malignancies and remains a major clinical challenge due to its high metastatic potential, heterogeneous antigenic landscape, and immune escape mechanisms (1). Immune checkpoint inhibitors, especially antibodies targeting the PD-1/PD-L1 axis, have substantially improved the therapeutic outcome of advanced melanoma (2). Despite the great clinical success, a substantial proportion of patients either fail to respond initially or eventually acquire resistance (3). The efficient antitumor immunity requires not only the activated cytotoxic T cells but also effective exposure and presentation of tumor-associated antigens, and sustained infiltration of cytotoxic T lymphocytes into the tumor microenvironment (4). Therefore, developing novel therapeutic strategies that simultaneously relieve immune suppression and enhance tumor immunogenicity may provide a promising approach for enhanced cancer immunotherapy (5).

The PD-1/PD-L1 axis plays a key role in inhibiting antitumor immunity (6). PD-L1 expressed on tumor cells or immunosuppressive cells selectively binds to PD-1 receptor on activated T cells, delivering inhibitory signals that reduce cell proliferation, cytokine secretion, and cytotoxic function, finally leading to T cell exhaustion (7). Immune checkpoint inhibitors can selectively disrupt the PD-1/PD-L1 axis and revive the exhausted T cells for tumor killing (8). Compared with antibody-based blockers, peptides offer potential advantages such as smaller molecular size, chemical synthesis, easier engineering, and flexible integration with delivery systems (9, 10). D-PPA1 peptide (NYSKPTDRQYHF), a D-peptide of PD-L1 antagonist, can selectively disrupt the PD-1/PD-L1 axis and elicit antitumor immunity for cancer immunotherapy (11, 12). Although D-PPA1 exhibits potent PD-L1 blockade activity, its clinical application in cancer therapy remains constrained by rapid systemic clearance, susceptibility to enzymatic degradation, and poor tumor delivery efficiency (13).

Increasing evidence has demonstrated that drug delivery systems can significantly improve the therapeutic efficacy of drugs with decreased side effects in healthy tissues (14, 15). Different from manmade nanocarriers, OMVs are natural nanovesicles released by Gram-negative bacteria for communication between cells (16, 17). Bacteria-derived OMVs can activate innate immune responses through pathogen-associated molecular patterns and intrinsic immunostimulatory activity (18–20). Recently, OMVs have also been developed as versatile platforms for delivering diverse therapeutic agents and nanoparticles for cancer treatment (21, 22) In addition, engineered OMVs can display tumor-associated antigens on their surface, thereby eliciting antigen-specific antitumor immune responses, highlighting their potential as a multifunctional platform for cancer therapy (23–25).

To date, efficient delivery of therapeutic peptides to target tissues remains a significant challenge (26). Conventional coupling strategies for peptide delivery often require complex chemical modifications and are frequently associated with low loading efficiency and limited yield (27). The emergence of the SpyCatcher/SpyTag system provides an efficient tool for the selective crosslinking of peptides/proteins on platforms (28, 29). SpyCatcher/SpyTag is a modular covalent coupling tool originated from the second immunoglobulin-like collagen adhesion domain (CnaB2) in the fibronectin binding protein FbaB of Streptococcus pyogenes (30). Owing to the high selectivity and efficiency of the ligation system, SpyTag-fused peptides or proteins can rapidly couple with SpyCatcher-based platforms, providing a modular strategy for the construction of personalized or multifunctional theranostic platforms (31, 32).

Many reports have shown that insufficient tumor antigen expression and presentation also contribute to the poor response of tumors to immunotherapy (33, 34). Accumulating studies have shown that epigenetic regulation in cancer cells is closely associated with tumor immune escape (35, 36). Zeb, a DNA methyltransferase (DNMT) inhibitor, has been reported to enhance tumor immunogenicity by activating cGAS-STING signal, promoting antigen processing and presentation, and enhancing the efficacy of cancer immunotherapy (37, 38). Therefore, DNMT inhibitor-based combination therapies have attracted increasing attention for enhancing cancer immunotherapy (39).

Leveraging immune checkpoint blockade and DNMT inhibitor-mediated enhancement of tumor immunogenicity, we developed a D-PPA1-modified bacterial OMV platform for zebularine delivery in melanoma therapy (OMV@PPA/Zeb). We hypothesized that D-PPA1 displayed on OMVs could block the PD-L1/PD-1 axis to promote T-cell activation, while Zeb could increase tumor-associated antigen expression and enhance antigen presentation. Through this dual mechanism, OMV@PPA/Zeb was expected to reprogram the immunosuppressive tumor microenvironment, promote the infiltration and activation of cytotoxic T cells, and ultimately enhance outcomes of cancer immunotherapy.

## 2 Materials and methods

### 2.1 Materials

Zeb, PE-antiPD-L1, antibodies targeting CD146, TRP1, and β-actin for western blot were obtained from Abcam. Ki67, calreticulin (CRT), and high mobility group box 1 protein, (HMGB1) antibodies were obtained from Proteintech. The SpyTag-DPPA1 peptide was synthesized by GL Biochem (Shanghai) LTD. Antibodies against CD3, CD4, CD8, perforin, granzyme B, and Foxp3 for flow cytometry analysis, interferon-gamma (IFN-γ) and tumor necrosis factor-alpha (TNF-α) ELISA kits were purchased from KeyGEN Biotech (China). CCK-8 kit, DAPI, and Dil were obtained from Beyotime (China).

### 2.2 Preparation and characterization of SpyCatcher-engineered OMVs

The gene encoding SpyCatcher003 was fused to the outer membrane protein OmpA to enable surface display of SpyCatcher on OMVs. The resulting recombinant plasmid was transformed into *E. coli* BL21(DE3) competent cells for OMV production. Engineered bacteria were cultured in LB medium until the optical density at 600 nm (OD600) reached 0.6–0.8, followed by induction with 0.5 mM IPTG for 16 h at 16 °C. The culture was then centrifuged at 5000 × g for 10 min at 4 °C to collect the supernatant. The supernatant was filtered through a 0.45 µm membrane and concentrated using a 100 kDa ultrafiltration tube, followed by a second filtration through a 0.22 µm membrane. Ultracentrifugation was subsequently performed at 150,000 × g for 3 h at 4 °C. The resulting OMV@Spycatcher were resuspended in pre-chilled PBS (pH 7.4), and their protein concentration was determined using a BCA protein assay kit. The presence of OmpA-SpyCatcher on OMVs was confirmed by western blotting. Size distribution and morphology were characterized using dynamic light scattering (DLS) and transmission electron microscopy (TEM), respectively.

### 2.3 Preparation of SpyTag-D-PPA1 peptide-decorated OMVs

To generate D-PPA1-decorated OMVs, a fusion peptide comprising SpyTag (RGVPHIVMVDAYKRYK) and D-PPA1 (D-{NYSKPTDRQYHF}) sequences was synthesized for conjugation. SpyCatcher-engineered OMVs were incubated with the SpyTag-D-PPA1 peptide at varying molar ratios, allowing the SpyTag/SpyCatcher reaction to proceed at room temperature for 30 min. Unbound SpyTag-D-PPA1 was removed via ultrafiltration. Successful conjugation of the fusion peptide onto OMVs was confirmed by western blotting, and the conjugation efficiency was determined by comparing the amount of SpyTag-D-PPA1 before and after purification.

### 2.4 Loading of Zeb into OMV@PPA

OMV@PPA/Zeb was prepared by incubating OMV@PPA with Zeb solution in a centrifuge tube. The mixture was placed in a beaker filled with ice and subjected to sonication for 30 min. The sample was then incubated on ice for 30 min to allow membrane recovery. Subsequently, the Zeb-loaded vesicles were collected by ultracentrifugation at 100,000 × g for 3 h. The resulting pellet constituted the Zeb-loaded OMVs. The loaded zeb into OMVs was determined by HPLC analysis. Drug-loading content (DLC) and encapsulation efficiency (EE) were calculated using the reported equations. DLC (%) = (Weight of drug in nanoparticles/Total weight of nanoparticles) × 100%. EE (%) = (Weight of drug in nanoparticles/Total weight of drug fed initially) × 100%. The hydrodynamic diameter and zeta potential of OMV@PPA/Zeb were measured by DLS. The morphology of nanoparticles was observed by TEM.

### 2.5 Cellular uptake of OMV@PPA

OMV@PPA were labeled with the fluorescent membrane dye DiI according to the manufacturer’s protocol. B16F10 melanoma cells were seeded in glass-bottom confocal dishes or plates suitable for flow cytometric analysis and incubated with DiI-labeled OMV@PPA for 2 h. After incubation, the cells were washed thoroughly with PBS to remove unbound vesicles. Cellular uptake was subsequently evaluated by confocal laser scanning microscopy (CLSM).

### 2.6 PD-L1 blockade assay

The PD-L1-binding activity of OMV@PPA was evaluated using a cell-based competitive binding assay. B16F10 melanoma cells were pretreated with OMV@SpyCatcher or OMV@PPA for 4 h. After removal of unbound vesicles by washing with PBS, the cells were incubated with a PE-conjugated anti-PD-L1 antibody for 1 h. The fluorescence intensity of antibody-bound cells was analyzed by flow cytometry in the PE channel.

### 2.7 CCK-8 assay

The biocompatibility of OMV@SpyCatcher was evaluated in HEK-293T cells using a Cell Counting Kit-8 (CCK-8) assay. HEK-293T cells were treated with increasing concentrations of OMV@SpyCatcher at 37°C for 48 h. Cell viability was then determined by measuring the absorbance at 450 nm.

The cytotoxicity of OMV@PPA/Zeb was assessed in B16F10 melanoma cells using the CCK-8 assay. Cells were seeded in 96-well plates and treated with PBS, OMVs, OMV@PPA, Zeb, OMV@Zeb, or OMV@PPA/Zeb at the indicated concentrations. After 48 h of incubation, cell viability was measured according to the manufacturer’s protocol.

### 2.8 Western blotting

Western blotting was performed to verify the presence of OmpA-SpyCatcher in engineered OMVs, assess the stability of OMV@SpyCatcher, and confirm the successful conjugation of the SpyTag-D-PPA1 peptide to OMV@SpyCatcher. These analyses were conducted using an anti-His antibody.

The effects of OMV@PPA/Zeb on the expression of melanoma-associated antigens and HMGB1 were also evaluated by western blotting in B16F10 cells and tumor tissues. Cells or tumor tissues were lysed in RIPA buffer supplemented with protease inhibitors. Equal amounts of protein were separated by SDS-PAGE and transferred onto poly(vinylidene difluoride) membranes. After blocking with nonfat milk, the membranes were incubated overnight at 4 °C with primary antibodies against His-tag, CD146, TRP1, and HMGB1. The membranes were subsequently incubated with the corresponding horseradish peroxidase-conjugated secondary antibodies, and protein bands were visualized using an enhanced chemiluminescence detection system.

### 2.9 *In vivo* antitumor assay

All animal experiments were conducted following the National Institutes of Health Guidelines for the Care and Use of Laboratory Animals, and the guidelines were approved by the Animal Protection and Utilization Committee of Taizhou University (Taizhou, Zhejiang). A subcutaneous melanoma model was established by injecting B16-F10 cells into the flank of C57BL/6 mice. When tumor volume reached approximately 50~80 mm³, mice were randomly divided into the following groups: PBS, OMVs (200µg per mouse), OMV@PPA (200µg per mouse), Zeb (20 mg/kg), OMV@Zeb (Zeb: 20 mg/kg), and OMV@PPA/Zeb (Zeb: 20 mg/kg). Mice were treated by intravenous injection according to the experimental schedule. Mice were injected with 200 µg of OMV protein and 20 mg/kg of Zeb every three days for a total of five treatments. Tumor volume and body weight were recorded every 3 days. Tumor volume was calculated using the following formula: Tumor volume = length × width²/2.

### 2.10 Flow cytometric analysis

Tumor tissues were minced and digested with collagenase, DNase I, and hyaluronidase to obtain single-cell suspensions. Cells were filtered through a cell strainer, washed, and blocked before staining. For immune profiling, cells were stained with antibodies against CD45, CD3, CD4, CD8, Foxp3, perforin, and granzyme B. Cytotoxic T cells were identified as CD3^+^CD8^+^ cells. Regulatory T cells (Treg) were identified as CD4^+^Foxp3^+^ cells. Data were acquired using a flow cytometer and analyzed with FlowJo software.

### 2.11 Histological, immunohistochemical, and immunofluorescence analysis

Tumors and major organs from different groups were fixed in 4% paraformaldehyde, embedded in paraffin, and sectioned. Hematoxylin and eosin (H&E) staining was performed to evaluate tissue morphology and systemic toxicity. Tumor apoptosis was analyzed by TUNEL staining. Ki67 staining was used to evaluate the tumor proliferation capability. CRT immunostaining was performed to assess the induced ICD effect. Immune cell infiltration was evaluated by immunofluorescent staining for CD8.

### 2.12 Serum biochemistry and biosafety evaluation

To evaluate systemic biosafety, blood samples were collected from treated mice from different groups. Serum biochemical indicators, including alanine aminotransferase (ALT), aspartate aminotransferase (AST), blood urea nitrogen (BUN), creatinine (CR), and uric acid (UA) levels, were measured using an automatic biochemical analyzer. Major organs, including the heart, liver, spleen, lung, and kidney, were collected for H&E staining. IFN-γ and TNF-α in serum were measured by ELISA assay.

### 2.13 Statistical analysis

All quantitative data are presented as mean ± standard deviation (SD). Statistical analyses were performed using SPSS software. Comparisons between two groups were conducted using an unpaired two-tailed Student’s t-test. For comparisons among multiple groups, one-way or two-way analysis of variance (ANOVA) followed by Tukey’s *post hoc* test was applied. A p-value of <0.05 was considered statistically significant. Statistical significance is indicated as follows: *P < 0.05, **P < 0.01, ***P < 0.001.

## 3 Results

### 3.1 Construction and characterization of SpyCatcher-engineered OMVs

**Figure 1:**
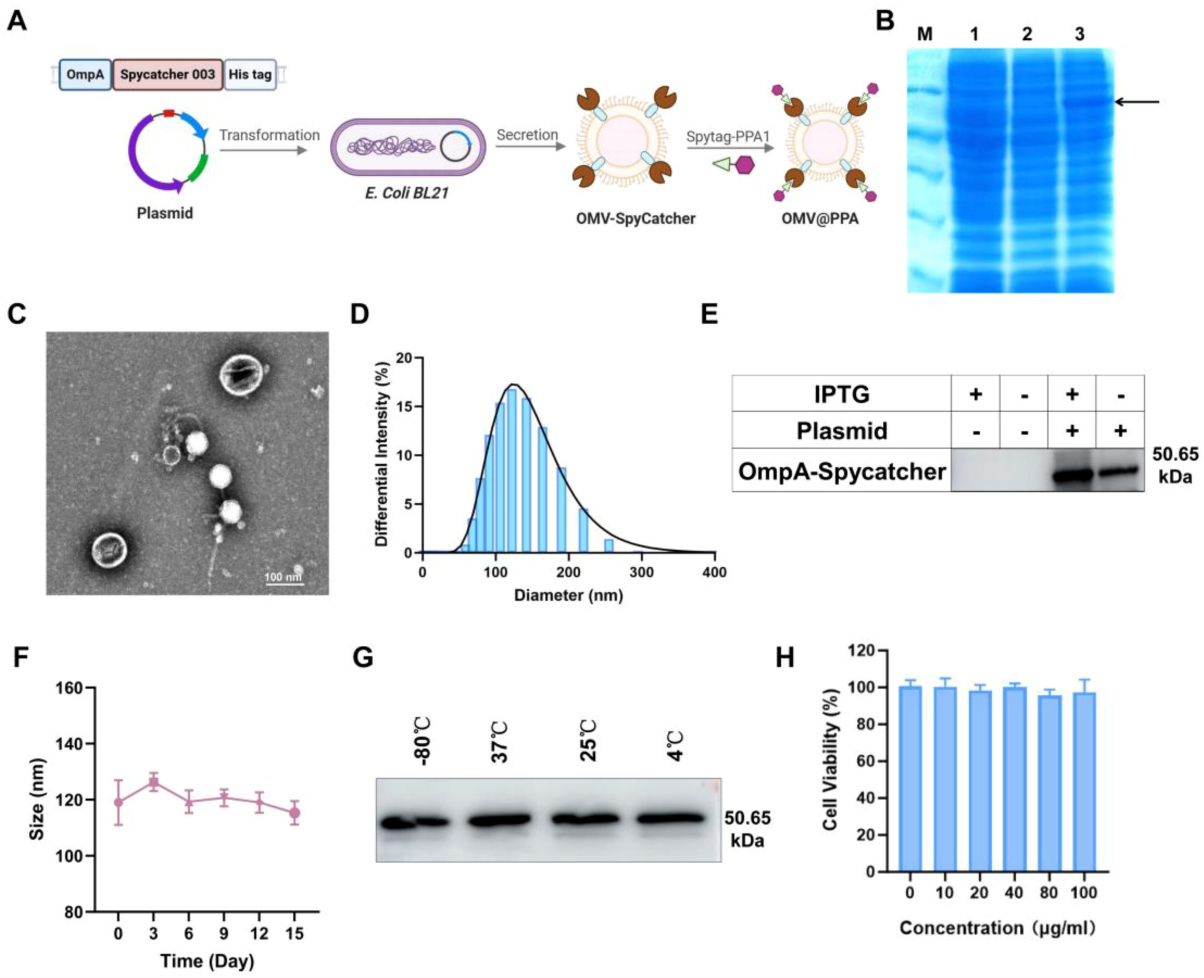
Characterization of OMV@SpyCatcher. **(A)** Schematic illustration of the preparation of OMV@SpyCatcher. **(B)** SDS-PAGE analysis of OmpA-SpyCatcher protein expression in the supernatant of different bacterial strains. M: protein marker; Lane 1: empty BL21; Lane 2: OmpA-SpyCatcher BL21 without IPTG induction; Lane 3: OmpA-SpyCatcher-engineered BL21 with IPTG induction. The arrow indicates the OmpA-SpyCatcher protein. **(C)** TEM analysis of OMV@SpyCatcher. Scale bar: 100 nm. **(D)** Particle size distribution analysis by DLS analysis. **(E)** Western blot analysis of OmpA-SpyCatcher in engineered OMVs using an anti-His antibody. **(F)** *In vitro* stability analysis of OMV@SpyCatcher by monitoring changes in vesicle size over time. OMV@SpyCatcher samples were stored at −20 °C for the indicated number of days (n=3). **(G)** Temperature-dependent stability of engineered OMVs under different incubation conditions. The vesicles were stored at the indicated temperatures for 24 h. **(H)** Biocompatibility assessment of OMV@SpyCatcher in HEK-293T cells using CCK-8 assay (n=4). HEK-293T cells were incubated with different concentrations of OMV@SpyCatcher at 37 °C for 48 h. Cell viability was then determined by measuring the absorbance at 450 nm.

In this study, to display SpyCatcher003 on the outer surface of OMVs, we fused the SpyCatcher003 sequence to the C-terminus of the membrane scaffold protein OmpA (**Figure 1A**). The sequenced plasmid was transformed into BL21 competent cells for engineered OMV preparation. The OmpA-SpyCatcher expression was confirmed through SDS-PAGE analysis (**Figure 1B**). OMV was isolated by the ultracentrifugation method and named OMV@SpyCatcher. TEM analysis revealed that the engineered OMVs retained a typical spherical vesicular morphology (**Figure 1C**). DLS analysis showed that OMV@SpyCatcher had a mean diameter of 122.0 nm (**Figure 1D**). Western blotting further confirmed the successful existence of OmpA-SpyCatcher on the engineered OMVs (**Figure 1E**). Size change analysis over 15 days of incubation, together with Western blot analysis of vesicle samples stored at different temperatures, demonstrated the excellent *in vitro* stability of OMV@SpyCatcher (**Figures 1F, G**). Biocompatibility was assessed by evaluating the viability of HEK-293T cells treated with different concentrations of OMV@SpyCatcher. CCK8 assay results confirmed the favorable compatibility of the engineered OMVs at tested concentrations (**Figure 1H**).

### 3.2 Preparation and characterization of OMV@PPA

**Figure 2:**
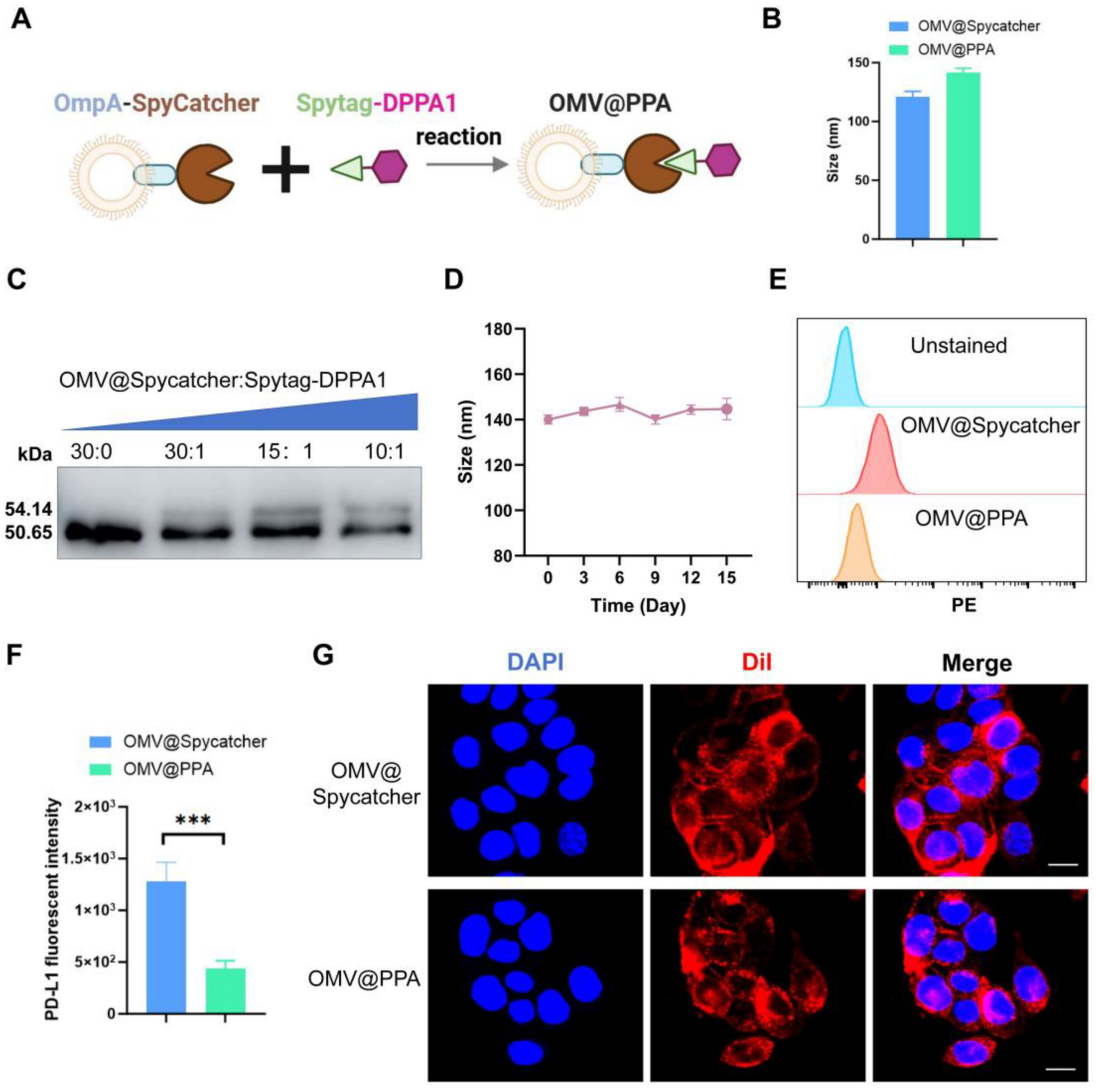
Characterization of D-PPA1 peptide-decorated OMVs (OMV@PPA). **(A)** Schematic illustration of ligation between OMV@SpyCatcher and SpyTag-DPPA1 peptide. **(B)** Particle size analysis of OMV@PPA (n=3). **(C)** Western blot demonstrated the successful decoration of the D-PPA1 peptide on engineered OMVs. The protein weight ratio of OMV@SpyCatcher to SpyTag-DPPA1 peptide ranged from 30:0 to 10:1. The upper band (54.14 kDa) indicates the OmpA-SpyCatcher–SpyTag-DPPA1 fusion protein, whereas the lower band (50.65 kDa) represents unreacted OmpA-SpyCatcher. **(D)** Stability analysis of OMV@PPA in PBS (pH 7.4) at 4 °C for different days. **(E)** PD-L1 blockade analysis of OMV@PPA in B16F10 cells. B16F10 cells were pretreated with equal concentrations (50 μg/mL) of OMV@SpyCatcher or OMV@PPA at 37 °C for 4 h. After washing three times with PBS, the cells were incubated with PE-conjugated anti-PD-L1 antibody (20 μg/mL) at 37 °C for 1 h. The treated cells were then detached with trypsin and subjected to flow cytometry analysis (gated on the PE channel). **(F)** Quantitative analysis of PD-L1 blockade based on mean fluorescence intensity (n=3). Statistical analysis was performed using one-way ANOVA followed by Tukey’s test with multiple comparisons. **(G)** Cellular uptake of DiI-labeled OMV@PPA in B16F10 cells. B16F10 cells were treated with DiI-labeled OMV@Spycatcher (100 μg/mL) or OMV@PPA (100 μg/mL) at 37 °C for 4 h. Blue: nucleus; red: DiI-labeled vesicles. ***p < 0.001.

To efficiently decorate D-PPA1 peptide on the surface of OMVs, we synthesized a SpyTag-DPPA1 fusion peptide containing both the SpyTag and DPPA1 sequences (**Supplementary Material**, **Supplementary Figure 1**). Due to the highly selective reaction between SpyCatcher003 and SpyTag (**Figure 2A**), the SpyTag-DPPA1 peptide can be efficiently conjugated to OMV@Spycatcher via the SpyTag/SpyCatcher system, resulting in the formation of OMV@PPA. DLS analysis revealed that D-PPA1 decoration increased the size by 20.1 nm (**Figure 2B**). The successful conjugation of D-PPA1 to OMVs was evaluated by Western blot. Increasing the ratio of SpyTag-DPPA1/OMV@SpyCatcher resulted in higher loading efficiency of D-PPA1 onto the vesicles (**Figure 2C**). OMV@PPA exhibited good *in vitro* stability, with no noticeable changes in particle size during 15 days of incubation (**Figure 2D**).

Given the role of DPPA1 in disrupting the PD-L1/PD-1 axis, we assessed whether OMV@PPA retained PD-L1-binding activity by measuring its inhibition of anti-PD-L1 antibody binding to melanoma cells. The melanoma cells were pretreated with OMV@PPA or OMV@Spycatcher for 4 hours, followed by staining with PE-labeled anti-PD-L1. Competitive binding assays demonstrated that pretreatment with OMV@PPA, but not OMVs@Spycatcher, significantly reduced the binding of anti-PD-L1 to B16F10 cells (**Figures 2E, F**), suggesting the effective PD-L1 blockade property of OMV@PPA. The internalization of OMV@PPA in B16F10 cells was assessed using Dil-labeled OMV@PPA. CLSM imaging revealed that OMV@PPA was efficiently internalized into the perinuclear region of the melanoma cells (**Figure 2G**), demonstrating good cellular uptake in melanoma cells.

### 3.3 OMV@PPA/Zeb enhances the expression of tumor-associated antigens in melanoma cells

**Figure 3:**
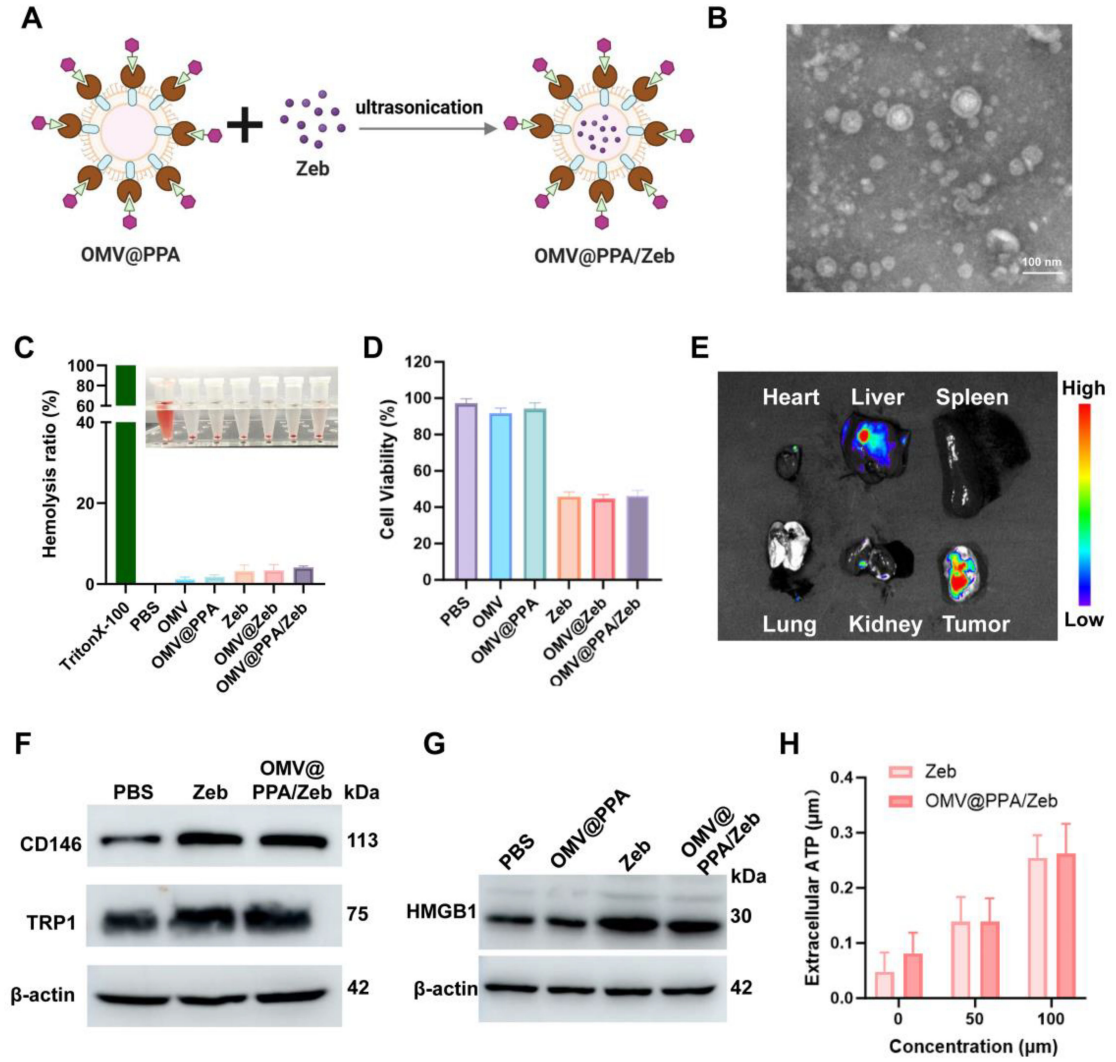
OMV@PPA/Zeb promotes the upregulation of melanoma-associated antigens and induces immunogenic cell death (ICD) in melanoma cells. **(A)** Schematic illustration of the preparation of OMV@PPA/Zeb. **(B)** Morphology analysis of OMV@PPA/Zeb via TEM imaging. Scale bar: 100 nm. **(C)** Hemolysis assay: representative images and quantitative analysis of different formulations (n=3). **(D)** Cytotoxicity of OMV@PPA/Zeb was evaluated using a CCK-8 assay (n=4). B16F10 cells were treated with PBS, OMV, OMV@PPA, free Zeb (200 μM), OMV@Zeb (equivalent Zeb concentration: 200 μM), or OMV@PPA/Zeb (equivalent Zeb concentration: 200 μM) for 24 h. **(E)** Biodistribution of OMV@PPA/Zeb in B16F10 tumor-bearing mice. DiR-labeled OMV@PPA/Zeb was intravenously injected into mice, and *in vivo* imaging was performed 48 h post-injection. **(F)** Western blot analysis of CD146 and TRP1 expression in B16F10 cells after different treatments. B16F10 cells were treated with free Zeb or OMV@PPA/Zeb at an equivalent Zeb concentration of 100 μM for 24 h. **(G)** HMGB1 expression in melanoma cells following OMV@PPA/Zeb treatment. B16F10 cells were treated with free Zeb or OMV@PPA/Zeb at an equivalent Zeb concentration of 100 μM for 48 h. **(H)** Extracellular ATP levels induced by free Zeb and OMV@PPA/Zeb (n=4). B16F10 cells were treated with free Zeb or OMV@PPA/Zeb at an equivalent Zeb concentration of 100 μM for 24 h.

To integrate epigenetic regulation into the vesicle platform, we loaded a DNMT inhibitor into OMV@PPA to generate an OMV-based codelivery system (OMV@PPA/Zeb). Zeb was loaded into the inner cavity of OMV@PPA via ultrasonication (**Figure 3A**). OMV@PPA/Zeb has a mean diameter of 164.0 nm (**Supplementary Material**, **Supplementary Figure 2**). TEM analysis confirmed the successful encapsulation of Zeb within the inner cavity of vesicles (**Figure 3B**). The drug loading capacity (DLC) and encapsulation efficiency (EE) for Zeb were determined to be 20.83 ± 0.76% and 26.40% ± 1.28%, respectively. Hemolysis analysis demonstrated the good biocompatibility of OMV@PPA/Zeb (**Figure 3C**). The cytotoxicity of the OMV@PPA/Zeb against melanoma cells was assessed using a CCK-8 assay. Compared to the no inhibitory effect of OMV@PPA on cell proliferation, Zeb and Zeb-loaded OMVs significantly suppressed the proliferation of B16F10 cells (**Figure 3D**). The *in vivo* biodistribution of OMV@PPA/Zeb was evaluated using fluorescence imaging. DiR-labeled OMV@PPA/Zeb was intravenously injected into B16F10 tumor-bearing mice, and fluorescence signals in tumors and major organs were analyzed 48 h post-injection. The results showed that OMV@PPA/Zeb predominantly accumulated in tumor tissues, with weaker signals observed in the liver and kidneys, supporting the tumor-targeting capability of the engineered OMV platform (**Figure 3E**).

We next assessed whether OMV@PPA/Zeb could enhance melanoma cell immunogenicity. Western blot analysis demonstrated that both free Zeb and OMV@PPA/Zeb markedly upregulated the expression of melanoma-associated antigens CD146 and TRP1 (**Figure 3F**, **Supplementary Material**, **Supplementary Figure 3**), indicating improved immune recognition after treatment with Zeb-loaded OMVs. Considering the potential of Zeb to promote ICD effect, we further examined whether OMV@PPA/Zeb could induce ICD in melanoma cells. Notably, treatment with either Zeb or OMV@PPA/Zeb markedly increased HMGB1 expression (**Figure 3G**, **Supplementary Material**, **Supplementary Figure 4**). In addition, OMV@PPA/Zeb enhanced extracellular ATP release (**Figure 3H**), further supporting its potent ICD-inducing activity in melanoma cells.

### 3.4 OMV@PPA/Zeb suppressed tumor growth in mice bearing B16F10 xenograft

**Figure 4:**
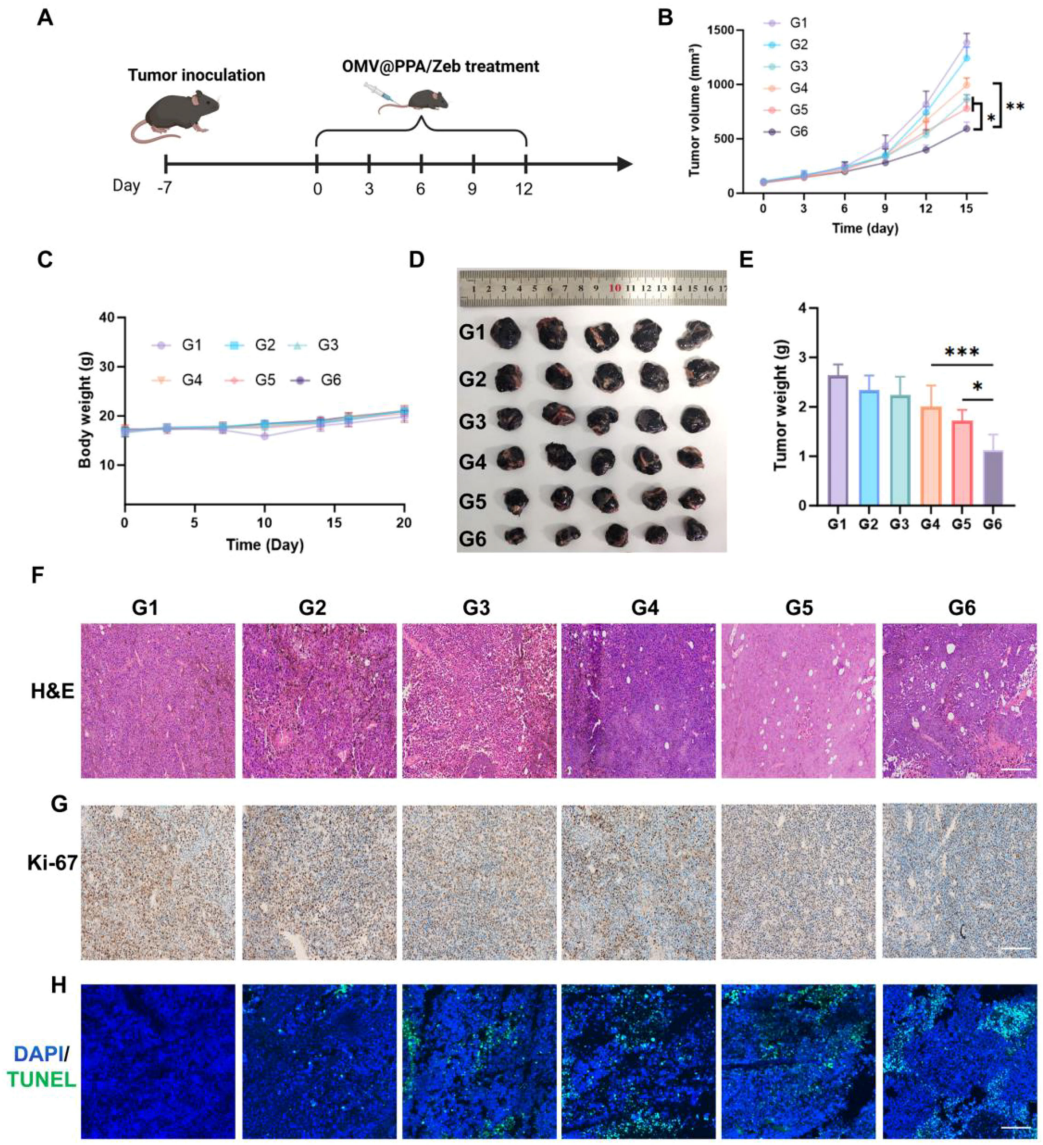
OMV@PPA/Zeb suppresses tumor growth in a B16F10 melanoma mouse model. Groups: G1, PBS; G2, OMV@Spycatcher; G3, OMV@PPA; G4, Zeb; G5, OMV@Zeb; G6, OMV@PPA/Zeb. **(A)** Schematic of tumor inoculation and treatment schedule. Mice were intravenously injected via the tail vein on the indicated days. B16F10 tumor-bearing mice were intravenously injected with PBS, OMVs (200 μg), OMV@PPA (200 μg), free Zeb (20 mg/kg), OMV@Zeb (equivalent Zeb dose: 20 mg/kg), or OMV@PPA/Zeb (equivalent Zeb dose: 20 mg/kg) every three days for a total of five treatments. **(B)** Tumor volume over time (n=5). **(C)** Body weight analysis (n=5). **(D)** Representative images of excised tumors on day 20. **(E)** Tumor weight analysis (n=5). **(F)** H&E staining of tumor sections in different groups. Scale bar: 100 μm. **(G)** Ki-67 immunostaining of tumor tissues following different treatments. Scale bar: 100 μm. **(H)** TUNEL assay of tumor sections. Scale bar: 100 μm. Data in panels B and E are presented as mean ± SD. Statistical significance was determined using one-way ANOVA followed by Tukey’s *post hoc* test. *p < 0.05, **p < 0.01, ***p < 0.001.

Based on the PD-L1 blockade activity and enhanced immunogenicity mediated by OMV@PPA/Zeb, we next evaluated its antitumor efficacy in a melanoma mouse model. Mice were randomly divided into six groups: PBS, OMV@Spycatcher, OMV@PPA, Zeb, OMV@Zeb, and OMV@PPA/Zeb. The mice received five intravenous injections of the indicated formulations on the scheduled treatment days (**Figure 4A**). Compared with the minimal tumor inhibition observed in the PBS and OMV@SpyCatcher groups, free Zeb, OMV@PPA, OMV@Zeb, and OMV@PPA/Zeb suppressed tumor growth to varying extents, with tumor inhibition rates of 28.3%, 37.8%, 43.8%, and 57.1%, respectively (**Figure 4B**). Among these formulations, OMV@PPA/Zeb exhibited the strongest tumor growth inhibition. The observed tumor inhibition rate of OMV@PPA/Zeb was 57.1%, which was slightly higher than the expected inhibition rate for the combination of Zeb and OMV@PPA calculated using the Bliss independence model (55.4%). This yielded a Bliss synergy score (ΔE) of 1.7%, indicating a mild synergistic effect under the tested dosing regimen. No obvious body weight loss was observed during the treatment period (**Figure 4C**), indicating good preliminary biosafety. Consistently, tumor weights were significantly reduced in the co-delivery system group (**Figures 4D, E**), further confirming the favorable *in vivo* therapeutic efficacy of OMV@PPA/Zeb.

The antitumor effect was further evaluated by histopathological staining. H&E staining of tumor sections revealed extensive tumor cell death in the OMV@PPA/Zeb group (**Figure 4F**). Ki-67 immunostaining showed markedly reduced proliferative activity after treatment with the combination formulation (**Figure 4G**). Consistently, TUNEL staining indicated that OMV@PPA/Zeb induced pronounced tumor cell apoptosis (**Figure 4H**). Collectively, these results confirmed the potent therapeutic efficacy of D-PPA1/Zeb-loaded OMVs.

### 3.5 OMV@PPA/Zeb enhances melanoma immunogenicity and induces ICD *in vivo*

**Figure 5:**
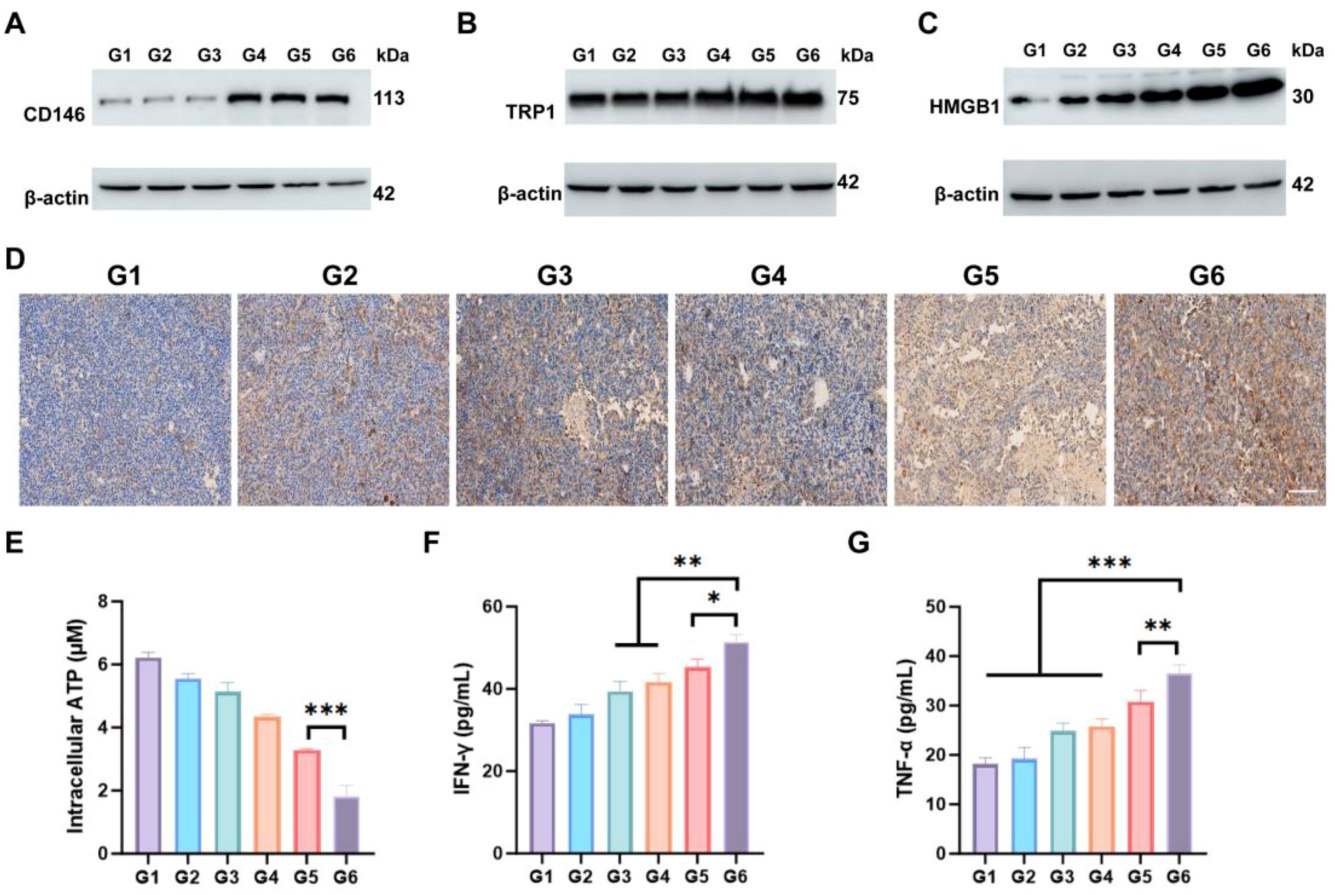
OMV@PPA/Zeb enhances the expression of melanoma-associated antigens and induces ICD effect *in vivo*. Groups: G1, PBS; G2, OMV@Spycatcher; G3, OMV@PPA; G4, Zeb; G5, OMV@Zeb; G6, OMV@PPA/Zeb. **(A)** Western blot analysis of CD146 expression in tumors from different groups. **(B)** TRP1 expression in tumors following treatment with OMV@PPA/Zeb. **(C)** Western blot analysis of HMGB1 expression in tumor tissues. **(D)** CRT staining in tumor sections. Scale bar: 100 μm. **(E)** Intratumoral ATP levels in different treatment groups (n=3). **(F)** Serum IFN-γ levels in treated mice (n=3). **(G)** Serum TNF-α levels in treated mice (n=3). Data in E~G are presented as mean ± SD. Statistical significance was determined using one-way ANOVA followed by Tukey’s *post hoc* test. ns: not significant; *p < 0.05, **p < 0.01, ***p < 0.001.

To clarify the immunoactivation mechanism, we examined melanoma-associated antigen expression in tumors following OMV@PPA/Zeb treatment. Western blot analysis revealed significant upregulation of CD146 and TRP1 in the OMV@PPA/Zeb group (**Figures 5A, B**, **Supplementary Material**, **Supplementary Figure 5**), indicating enhanced immune recognition of melanoma cells. The ICD-inducing effect of OMV@PPA/Zeb was also evaluated *in vivo*. OMV@PPA/Zeb markedly increased HMGB1 expression (**Figure 5C**, **Supplementary Material**, **Supplementary Figure 6**) and promoted CRT exposure in tumor tissues (**Figure 5D**). Moreover, the decreased intracellular ATP level was consistent with ATP release triggered by OMV@PPA/Zeb (**Figure 5E**), further supporting its ability to activate antitumor immunity. Moreover, the serum levels of pro-inflammatory cytokines induced by OMV@PPA/Zeb were evaluated by ELISA. Notably, OMV@PPA/Zeb induced a 1.63-fold increase in IFN-γ (**Figure 5F**) and a 2.01-fold increase in TNF-α levels (**Figure 5G**) compared with the PBS group, demonstrating the elicited antitumor immune response.

### 3.6 OMV@PPA/Zeb elicits a robust antitumor immune response *in vivo*

**Figure 6:**
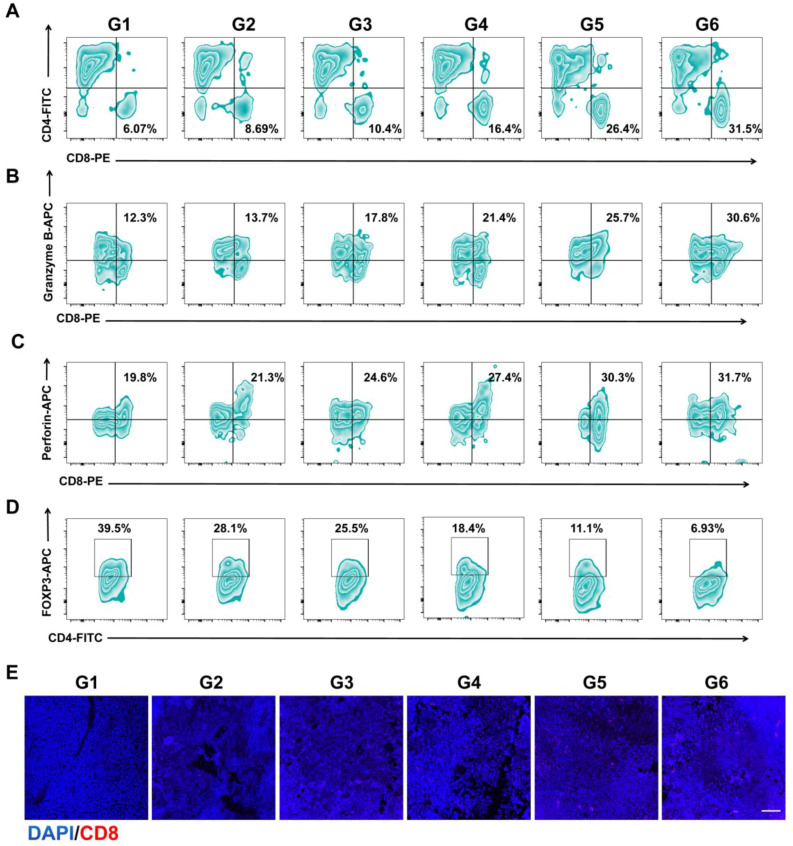
OMV@PPA/Zeb elicited a robust antitumor immune response *in vivo*. Groups: G1, PBS; G2, OMV@Spycatcher; G3, OMV@PPA; G4, Zeb; G5, OMV@Zeb; G6, OMV@PPA/Zeb. **(A)** Representative flow cytometry images showing CD8^+^ T cell infiltration in tumors. **(B)** Representative images showing CD8^+^GranzymeB^+^ T cell infiltration in tumor tissues by flow cytometry. **(C)** Representative images showing the percentage of CD8^+^Perforin^+^ T cells in tumors by flow cytometry. **(D)** Flow cytometry analysis of regulatory T (Treg) cells within the tumor microenvironment. **(E)** Immunohistochemical staining of CD8^+^ T cell infiltration in tumor sections across treatment groups. PE-labeled anti-CD8 antibody was used to track the distribution of CD8^+^ T cells. Blue: nucleus; Red: CD8^+^ T cells. Scale bar: 100 μm.

To elucidate the immune mechanism underlying the antitumor efficacy, tumor-infiltrating immune cells were analyzed by flow cytometry and immunofluorescence staining. Flow cytometry analysis showed that OMV@PPA/Zeb increased intratumoral infiltration of CD8^+^ T cells by 5.26-fold compared with PBS treatment (**Figure 6A**, **Supplementary Material**, **Supplementary Figure 7A**). Moreover, the proportions of perforin-positive CD8^+^ T cells (**Figure 6B**; **Supplementary Figure 7B**) and granzyme B-positive CD8^+^ T cells (**Figure 6C**; **Supplementary Figure 7C**) were significantly increased by 1.89- and 3.04-fold, respectively, indicating enhanced CD8^+^ T-cell activation. Moreover, OMV@PPA/Zeb substantially decreased the proportion of Tregs within the tumor microenvironment compared with the control groups (**Figure 6D**, **Supplementary Material**, **Supplementary Figure 7D**). Consistently, immunofluorescence staining further confirmed enhanced CD8^+^ T cell infiltration in tumor tissues from the OMV@PPA/Zeb group (**Figure 6E**). Collectively, these results suggest that the OMV@PPA/Zeb remodels the melanoma immune microenvironment from an immunosuppressive state toward an immune-activated phenotype.

**Figure 7:**
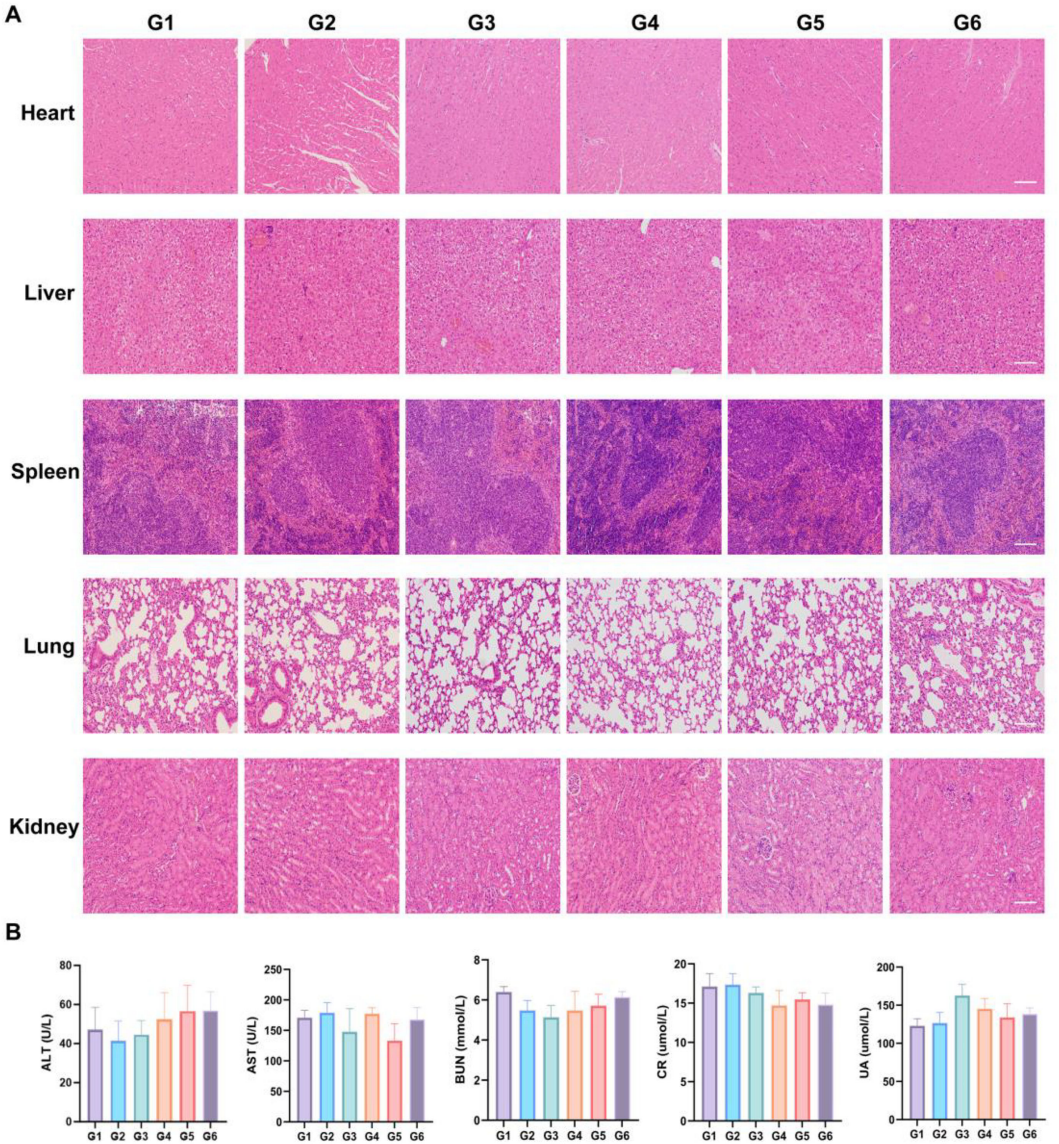
*In vivo* biosafety evaluation of OMV@PPA/Zeb. Groups: G1, PBS; G2, OMVs; G3, OMV@PPA; G4, Zeb; G5, OMV@Zeb; G6, OMV@PPA/Zeb. **(A)** H&E staining of major organs (heart, liver, spleen, lung, and kidney) and tumor tissues from mice in different treatment groups. **(B)** Serum biochemical analysis of ALT, AST, BUN, CR, and UA levels in mice receiving different treatments (n=3).

Finally, we evaluated the biosafety of OMV@PPA/Zeb in a melanoma-bearing mouse model. H&E staining of major organs, including the heart, liver, spleen, lung, and kidney, revealed no apparent histopathological abnormalities or tissue damage (**Figure 7A**). Liver and kidney functions were further assessed by measuring serum ALT, AST, BUN, Cr, and UA levels across different treatment groups. These results demonstrated the favorable *in vivo* biosafety profile of OMV@PPA/Zeb (**Figure 7B**).

## 4 Discussion

In this study, we developed a SpyCatcher-engineered bacterial OMVs system for the co-delivery of D-PPA1 peptide and DNMT inhibitor to achieve combined melanoma immunotherapy. Our results demonstrate that OMV@PPA/Zeb can efficiently activate the antitumor immune response and significantly inhibit melanoma growth *in vivo*.

SpyTag/SpyCatcher-mediated modular assembly is used in this study for OMV@PPA/Zeb preparation (40). This modular design improves flexibility and may allow rapid substitution of different peptides. Previous studies have demonstrated that engineered OMVs can display tumor antigens and induce antigen-specific T-cell responses (23); our study extends this concept by using OMVs not only as antigen or peptide carriers but also as a multifunctional immunotherapeutic platform. Given the key role of the PD-L1/PD-1 axis in mediating tumor immune escape, disruption of this interaction can restore T-cell-mediated tumor killing. In our system, displaying D-PPA1 on OMVs confers checkpoint blockade activity and triggers an antitumor immune response. Compared with systemic antibody therapy, peptide-decorated OMVs may offer a more flexible platform for combination delivery.

Tumors frequently evade immune recognition by reducing antigen expression or impairing antigen processing and presentation (41). Zeb can enhance tumor immunogenicity and promote antigen processing and presentation machinery, mediating effective immunotherapy through the cGAS-STING pathway (37). In our study, DNMT inhibitor-loaded OMVs increased the expression of tumor-associated antigens and triggered immune cell death *in vitro* and *in vivo*, thereby providing a stronger antigenic basis for immune recognition.

The good antitumor effect of OMV@PPA/Zeb likely results from a coordinated immune activation process. This strategy addresses two major limitations of melanoma immunotherapy: insufficient tumor immunogenicity and immune checkpoint-mediated T-cell dysfunction. Zeb increases tumor antigen availability and antigen presentation. D-PPA1 peptide revives the exhausted T cells for immune killing. These effects together enhance T-cell priming, infiltration, and cytotoxic function.

## 5 Conclusion

In conclusion, we developed SpyCatcher-engineered OMVs loaded with D-PPA1 peptide and Zeb for combination immunotherapy in melanoma. By combining PD-L1/PD-1 checkpoint blockade with epigenetic enhancement of tumor antigen expression and presentation, this system effectively remodels the tumor immune microenvironment and promotes robust antitumor immunity. This work offers a promising strategy for the development of modular OMV-based combination immunotherapies for melanoma and potentially other solid tumors.

## Data Availability

The original contributions presented in the study are included in the article/**Supplementary Material**. Further inquiries can be directed to the corresponding authors.

## Supplementary material

Scheme 1 Schematic illustration of the mechanism of OMV@PPA/Zeb in triggering antitumor immune response for enhanced immunotherapy.
