# Supplementary material for: Modular engineered outer membrane vesicles for synergistic cancer immunotherapy via PD-L1 blockade and epigenetic reprogramming
Source: Front Immunol. 2026 Jul 31;17:1915499. doi: 10.3389/fimmu.2026.1915499 (PMC13473442; doi:10.3389/fimmu.2026.1915499)
Supplement: Supplementary file 1 [file Table1.doc]

**Supplementary Material**

**Modular Engineered Outer Membrane Vesicles for Synergistic Cancer Immunotherapy via PD-L1 Blockade and Epigenetic Reprogramming**

Longxue Guan^1,2,a^, Qinxiao Sun^2,a^, Dandan Liang^2^, Zhimin Li^2^, Shiqi Zhang^2^, Laili Huang^2^, Xingang Guan^1,*^, Hongxia Feng^1,*^

^1^Department of Dermatology, The Affiliated Wenling Hospital of Taizhou University (The First People’s Hospital of Wenling), School of Medicine, Taizhou University, Taizhou 317500, PR China

^2^College of Medical Technology, Beihua University, Jilin 132013, P. R. China

^a^These authors contribute to this work equally.

**Corresponding author:** guanxg@tzc.edu.cn (Guan X); fenghongxia0472@163.com (Feng H).

**Figure S1. The chemical structural formula of SpyTag-D-PPA1 peptide.**

**Figure S2. Size distribution analysis of OMV@PPA/Zeb by DLS.**

**Figure S3. Uncropped western blot images of CD146 and TRP1 expression in B16F10 cells treated with OMV@PPA/Zeb.** M: Marker; Lane 1: PBS; Lane 2: Zeb; Lane 3: OMV@PPA/Zeb.

**Figure S4. Uncropped western blot images of HMGB1 expression in B16F10 cells treated with OMV@PPA/Zeb.** M: Marker; Lane1:PBS; Lane2: OMV@PPA; Lane3: Zeb; Lane4: OMV@PPA/Zeb.

**Figure S5. Uncropped western blot images of CD146 and TRP1 expression in tumor tissues treated with different formulations.** M: Marker; Lane 1: PBS; Lane 2: OMV; Lane 3: OMV@PPA; Lane 4: Zeb; Lane 5: OMV@Zeb; Lane 6: OMV@PPA/Zeb.

**Figure S6. Uncropped western blot images of HMGB1 expression in in tumor tissues treated with different formulations.** M: Marker; Lane 1: PBS; Lane 2: OMV; Lane 3: OMV@PPA; Lane 4: Zeb; Lane 5: OMV@Zeb; Lane 6: OMV@PPA/Zeb.

**Figure S7. Quantitative analysis of immune cell infiltration in tumors by flow cytometry.** G1, PBS; G2, OMV@Spycatcher; G3, OMV@PPA; G4, Zeb; G5, OMV@Zeb; G6, OMV@PPA/Zeb. (A) Intratumoral CD8^+^ T cell infiltration. (B) Proportion of perforin^+^CD8^+^ T cells in tumors. (C) Proportion of granzyme B^+^CD8^+^ T cells in tumors. (D) Regulatory T cell infiltration within the tumor microenvironment. Data are presented as mean ± SD. Statistical significance was determined using one-way ANOVA followed by Tukey’s post hoc test. *p < 0.05, **p < 0.01, ***p < 0.001.
